# Red nucleus and rubrospinal tract disorganization in the absence of *Pou4f1*

**DOI:** 10.3389/fnana.2015.00008

**Published:** 2015-02-05

**Authors:** Jesus E. Martinez-Lopez, Juan A. Moreno-Bravo, M. Pilar Madrigal, Salvador Martinez, Eduardo Puelles

**Affiliations:** ^1^Instituto de Neurociencias de Alicante, Universidad Miguel Hernandez, Consejo Superior de Investigaciones Científicas (UMH-CSIC)San Juan de Alicante, Alicante, Spain; ^2^Instituto Murciano de Investigación Biomédica Virgen de la Arrixaca IMIB-Arrixaca, Universidad de MurciaMurcia, Spain

**Keywords:** midbrain, red nucleus, rubrospinal tract, *Pou4f1*, development, maturation, *Cplx1*, *Npas1*

## Abstract

The red nucleus (RN) is a neuronal population that plays an important role in forelimb motor control and locomotion. Histologically it is subdivided into two subpopulations, the parvocellular RN (pRN) located in the diencephalon and the magnocellular RN (mRN) in the mesencephalon. The RN integrates signals from motor cortex and cerebellum and projects to spinal cord interneurons and motor neurons through the rubrospinal tract (RST). *Pou4f1* is a transcription factor highly expressed in this nucleus that has been related to its specification. Here we profoundly analyzed consequences of *Pou4f1* loss-of-function in development, maturation and axonal projection of the RN. Surprisingly, RN neurons are specified and maintained in the mutant, no cell death was detected. Nevertheless, the nucleus appeared disorganized with a strong delay in radial migration and with a wider neuronal distribution; the neurons did not form a compacted population as they do in controls, *Robo1* and *Slit2* were miss-expressed. *Cplx1* and *Npas1*, expressed in the RN, are transcription factors involved in neurotransmitter release, neuronal maturation and motor function processes among others. In our mutant mice, both transcription factors are lost, suggesting an abnormal maturation of the RN. The resulting altered nucleus occupied a wider territory. Finally, we examined RST development and found that the RN neurons were able to project to the spinal cord but their axons appeared defasciculated. These data suggest that *Pou4f1* is necessary for the maturation of RN neurons but not for their specification and maintenance.

## Introduction

The red nucleus (RN) is a compacted neuronal population that plays an important role in motor control and locomotion. Its origin is attributed to limb development in vertebrate tetrapods and its histological structure changes considerably during mammalian development (Massion, 1967; ten Donkelaar, 1988; Gruber and Gould, 2010). This population is organized in two subnuclei. The parvocellular RN (pRN) is located in the caudal diencephalic basal plate. It is primarily compound by small and scattered large neurons. It contains mixed GABAergic and glutamatergic cells. This subnucleus continues caudally as the magnocellular RN (mRN) located in the basal midbrain. It is comprised mainly by large neurons that are exclusively glutamatergic (Gruber and Gould, 2010; Liang et al., 2012a,b; Moreno-Bravo et al., 2012; Puelles et al., 2012). These neurons receive motor system inputs from the cerebral cortex and the cerebellum. The processed information is transmitted through projections to spinal cord inter- and motor neurons (Nyberg-Hansen and Brodal, 1964; Nyberg-Hansen, 1966; Massion, 1967; Warner and Watson, 1972; Wild et al., 1979; Holstege, 1987; Holstege et al., 1988; Küchler et al., 2002; Liang et al., 2012a,b). The RN projection to the spinal cord, the rubrospinal tract (RST), crosses the midline in the ventral tegmental decussation (vtg) located in the caudal midbrain. After that, the RST forms a contralateral tract in the dorsolateral corner of the lateral funiculus with 97% *vGluT2* positive neurons (Du Beau et al., 2012; Liang et al., 2012a,b; Watson and Harrison, 2012). The importance of this system resides in its role in establishing rudimental motor skills that subsequently become refined by further and direct corticospinal control (Williams et al., 2014).

In the last years, unveiling genetic mechanisms that underlie neuronal differentiation has been of general interest. This differentiation involves two major aspects. On the one hand, a complex signaling process by morphogenes secreted from secondary organizers (Ruiz-i-Altaba, 1998; Martínez, 2001; Echevarría et al., 2003; Vieira et al., 2010). On the other hand, different genetic cascades that are triggered in neuronal precursors to direct their specification by signaling processes. These genetic cascades are composed by transcription factors that sequentially generate a precise differentiation pathway.

With the aim to broaden our knowledge of the RN differentiation program, we identified *Pou4f1* as a firm candidate playing a role in this process. It is an important transcription factor with a DNA binding POU domain. Its role in sensory peripheral nervous system development has been deeply studied (Gerrero et al., 1993; Fedtsova and Turner, 1995; Turner et al., 1996; Xiang et al., 1997; Trieu et al., 1999; Eng et al., 2001). In the central nervous system, its role has been well analyzed in tectum, habenula and retina (Eng et al., 2001; Mu et al., 2004; Fedtsova et al., 2008; Quina et al., 2009; Dykes et al., 2011; Badea et al., 2012). In the RN, *Pou4f1*^−/−^ neurons fail to survive and newborns show behavioral defects (Fedtsova and Turner, 1995; McEvilly et al., 1996; Xiang et al., 1996; Agarwala and Ragsdale, 2002). Other loss-of-function studies in sensory axon growth have shown that affected neurons undergo apoptosis and fail to correctly innervate their peripheral targets (Eng et al., 2001).

Our aim was to further analyze the role of *Pou4f1* in RN development and maturation. We selected *Npas1* and *Cplx1* to study RN neuron maturation. *Npas1*, member of the basic helix-loop-helix PAS (bHLH-PAS) family, is involved in neurogenesis and the transition from a proliferative to a differentiative state (Erbel-Sieler et al., 2004; Ohsawa et al., 2005; Sha et al., 2012). *Cplx1* is a small, hydrophilic protein that binds reversibly to the SNARE complex and modulates synaptic vesicle release. It is essential for normal motor function and performance of other complex behaviors (Glynn et al., 2005, 2007; Webster et al., 2011; Kielar et al., 2012). Due to the above mentioned, we consider them valuable markers of RN neuronal maturity. We also analyzed guidance molecules to describe possible reasons for the altered migration and final distribution of the RN neurons. We also analyzed *Caspase3*, to corroborate early descriptions of RN apoptosis in late stages of embryonic development (McEvilly et al., 1996; Xiang et al., 1996), but no cell death was detected.

Our data corroborate that *Pou4f1* is necessary for the correct maturation of the RN neurons, but not for the specification and maintenance of these neurons.

## Materials and methods

### Animals

The transgenic mice *Pou4f1^TauLacZ/+^* was generated as previously described (Quina et al., 2005). The day when the vaginal plug was detected was considered as embryonic day 0.5 (E0.5). Embryos were fixed in PBS 1x (NaCl 13 mM, Sigma S3014; KCl 0.3 mM, Sigma P9541; Na_2_HPO_4_ 1 mM, Sigma S3264 and KH_2_PO_4_ 0.2 mM, Sigma P9791) with 4% paraformaldehyde (PFA, Panreac 141451.1211) overnight at 4°C. Embryos were washed in PBS 1x, embedded in 4% agarose (Pronadisa 8008) and sectioned in 100 μm vibratome sections. For wax embedded sections, embryos were completely dehydrated, washed twice in 100% butanol (Panreac 14.682.1211) wax embedded (Gemcut emerald paraffin, Spiele no. 24364-1) and then sectioned in parallel series (7 μm). All mouse experiments were performed according to protocols approved by the Universidad Miguel Hernandez CEIE committee (ref. INA-EP001-10).

### *In situ* hybridization

The sections were dewaxed at 65°C and completely rehydrated. To facilitate probe penetration, tissue was incubated with proteinase K (0.01 mg/ml) in PBS-T (PBS 1x with 0.1% tween 20, Sigma P1379) and post fixed in 4% PFA. The sections were washed in PBS-T and prehybridized for 1 h in hybridization buffer comprised of 50% deionized formamide (Amresco, 0606), SALT 1X (NaCl 0.2 M, Sigma S3014, tris-HCl 9 mM Sigma T3253, Tris-Base 1 mM, Sigma T6066, NaH_2_PO_4_·2H_2_O 5 mM, Scharlau SO0334, Na_2_HPO_4_ 5 mM, Sigma S3264 and EDTA 5 mM, Sigma E5134) Denharts 2X (Bio Basic Canada D0062), Dextran sulfate 0.2 mM (Amresco, 0198) and 0.1% ARNt (Sigma R6625). The RNA probes were obtained from Source Bioscience/ ImaGenes (*Cplx1*, IRAVp968A0151D and *Npas1*, IRCKp5014E128Q) or construction kindly provided by Dr. O. Marin (*Robo1* and *Slit2*). These digoxigenin-labeled RNA probes (DIG-11-UTP, Roche Diagnostics, 11209256910) were denaturalized at 80°C and incubated with the tissue in hybridization buffer overnight at 62°C. The next day sections were washed in wash solution with 50% SSC 1x pH 7 (NaCl 0.15 M, Na_3_C_6_H_5_O_7_·2H_2_O 15 mM, Sigma C8532), 25% formamide (Sigma, F7503) and 0.1% tween 20 at 65°C and incubated with MABT 1x pH 7.4 (NaCl 40 mM, maleic acid 20 mM, NaOH 40 mM and 0.1% tween 20) with 10% sheep serum (Sigma, S2263) and 20% blocking reagent (Roche, 10057177103). After blocking, tissue was incubated overnight at 4°C in the same solution with an alkaline anti-digoxigenin antibody (1:3500, Roche, 11093274910). Excess of non-specific anti-digoxigenin antibody was extensively washed in MABT. Finally, the sections were washed with NTMT (NaCl 0.1 M, Sigma S3014, Tris-HCl 0.1 M pH 9.5, Sigma T3253, MgCl2·6H_2_O 0.05 M, VWR 1.05833 and 0.1% tween-20) and incubated overnight at room temperature in NTMT with 0.45 μl/ml of 4-Nitro blue tetrazolium chloride (NBT, 75 mg/ml, Roche, 70227721) and 3.5 μl/ml of 5-Bromo-4-Chloro-3-indolyl-phosphate, (BCIP, 50 mg/ml, Roche 11585002001). The NBT/BCIP was used for the colorimetric reaction to detect the presence of the hybridized probes. The alkaline phosphatase reacts with these substrates and produces a solid blue precipitate.

### Immunohistochemistry

The sections were dewaxed, completely rehydrated and for antigen retrieval boiled in sodium citrate 0.1 M pH 6. The sections were washed in phosphate buffer solution (PBT, Na_2_HPO_4_·12H_2_O 0.8 M, Panreac 141678.1214, NaCl 0.15 M, Panreac 121659.1211 and 0.075% Triton-X100, Sigma X100) and incubated in PBT with 1.5% H_2_O_2_ for 30 min to inactivate endogenous peroxidase. After inactivation, tissue was washed in PBT and blocked 1 h in PBT with 0.1% albumin bovine serum (BSA, A2153, Sigma) and 10% lysine 1 M (L5626, Sigma). Next, sections were incubated overnight at room temperature in PBT with 0.1% BSA and 0.01% sodium azide (S2002, Sigma) with different antibodies: αCaspase3 (1:300, Cell Signaling #9661), αChAT (1:100, Chemicon AB144P), αβGalactosidase (1:500, Abcam ab9361) and αTH (1:1000, Inst.J.Boy 28020234). The day after, the tissue was rinsed in PBT and incubated 1 h with the appropriate biotinylated secondary antibody at 1:200. Afterwards, the sections were washed in PBT and incubated in PBT with Avidin–Biotin Complex (1:500, Vectastain PK-4000) for 1 h. Finally, tissue was washed in PBT and Tris 0.1 M pH 7 and the immunolabeling was revealed in Tris 0.1 M with 1% 3-3’ diaminobenzidine tetrahydroc (DAB, Acros Organics W0572M) and 0.003% H_2_O_2_ leading to a brown precipitate.

### X-gal stainning

Fresh tissue from heterozygous or mutant mice was fixed in PBS 1x with 2% PFA for maximum 10 min. The reaction was made overnight at 37°C in X-gal staining solution (x-gal 1 mg/ml, Stratagene 200384-5, potassium hexacyaoferrate 20 mM, Prolabo 26 810.298, potassium ferrocyanide 20 mM, Prolabo, 26 816.298, magnesium chloride 2 mM, Prolabo 28 109.298, EGTA 5 mM, Sigma E3889, 0.01% sodium deoxycholate, Sigma D6750 and 0.02% NP-40, Sigma I3021) leading to a blue color.

## Results

### Aberrant generation of the red nucleus

**Figure 1:**
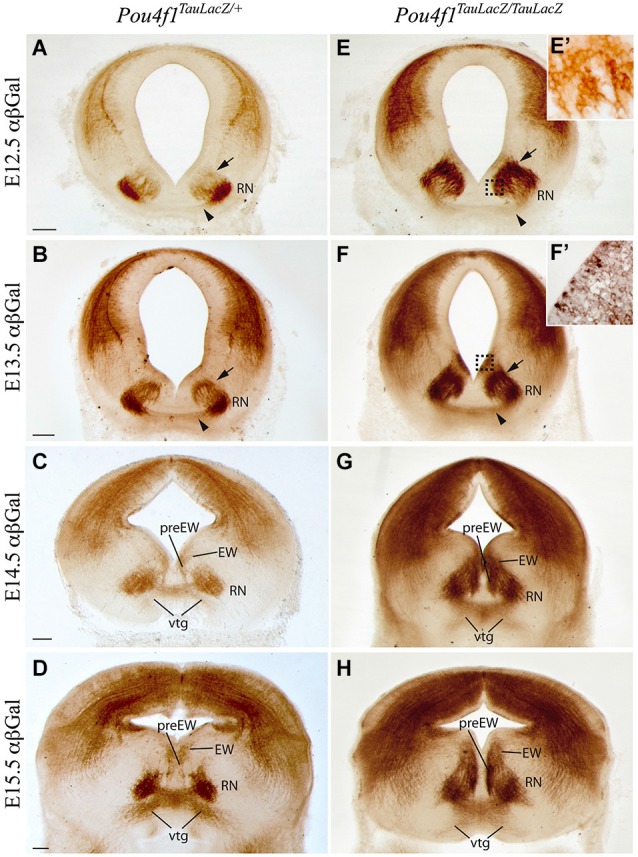
**Development of RN and RST in absence of *Pou4f1***. Coronal mesencephalic sections in *Pou4f^TauLacZ/+^* **(A–D)** and *Pou4f^TauLacZ/TauLacZ^* **(E–H)** embryos processed by immunohistochemistry. We have analyzed the embryonic stages: E12.5 **(A,E,E’)**, E13.5 **(B,F,F’)**, E14.5 **(C,G)** and E15.5 **(D,H)**. We followed the normal generation of the RN and its projections in the control embryos. The RN development in the mutant embryos showed a clear delay in radial migration. The RN displayed certain spatial disorganization. Squares in **(E)** and **(F)** shows magnification of these areas in **(E’)** and **(F’)** respectively. The arrowhead points to the rubrospinal tract. The arrow points to migrating neurons. Abbreviations: EW, Edinger-Westphal nucleus; RN, red nucleus; preEW, pre-Edinger-Westphal; vtg, ventral tegmental decussation. Scale bars = 150 μm.

The *Pou4f1^TauLacZ/+^* strain was used as a tool to label the RN neurons and its projections. We followed the embryonic development of the parvocellular and magnocellular subpopulations from E12.5 until E15.5, when these basal midbrain populations are well stablished. In the heterozygous mice at E12.5, we could already identify the RN in the mantle layer of the mesencephalic basal medial domain (Figure 1A). Scattered positive neurons migrated radially from the ventricular layer (arrow in Figure 1A) to their final destination. At this stage, the first positive axonal fibers of the RST were detected (arrowhead in Figure 1A). At E13.5, a big number of positive neurons remained migrating radially from the ventricular layer (arrow in Figure 1B). A dense group of positive axons were detected in the midline of the floor plate (arrowhead in Figure 1B). One day later, at E14.5, almost all the RN neurons were located at their final destination and organized in a compacted neuronal population. Positive axons crossed the midline, generating the vtg, and were navigating to the contralateral side of the neural tube (Figure 1C). Medial to the presumptive area of the oculomotor nucleus appeared a small population of *Pou4f1* positive neurons that correspond to the Edinger-Westphal and pre-Edinger-Westphal nuclei (Figure 1C). At E15.5, the RN and the first part of the RST were completely developed (Figure 1D).

We analyzed the development of this nucleus in the *Pou4f1* loss-of-function embryos (*Pou4f1^TauLacZ/TauLacZ^*). At E12.5, LacZ- positive neurons were located closer to the ventricular layer (arrow in Figures 1E,E’). Just a few of them were located in the mantle layer (Figure 1E). However, the first positive axons of the RST were observed at this stage (arrowhead in Figure 1E). At E13.5, the number of LacZ- positive cells in the mantle layer was increased but there were still many RN neurons migrating radially (arrow in Figure 1F). Surprisingly, the corresponding ventricular layer appeared also positive. A closer view allowed us to confirm the presence of positive cells in this layer (Figure 1F’), another sign of radial migration alterations. The RST, as in the control, was located in the midline of the floor plate (arrowhead in Figure 1F). At E14.5, the vtg was clearly visible (Figure 1G) and the nucleus was disorganized and did not display a compacted composition. The Edinger-Westphal and pre-Edinger-Westphal nuclei were more densely stained and occupied a wider territory (Figure 1G). The ventricular layer was still positive. Finally, at E15.5 the RN showed a wider neuronal distribution as compared to the control. Nevertheless, the first segment of the RST was generated and did not display any obvious alteration (Figure 1H). The ventricular layer appeared now negative for our marker (Figure 1H).

In summary, the RN neurons are specified despite the absence of *Pou4f1* but they are not able to generate a proper RN. However, the development of the RST first segment is unaltered.

### Altered maturation of the red nucleus

With the aim to study the final development of RN neurons, we analyzed control and mutant embryos at E18.5. We independently studied the rostral (pRN) and the caudal (mRN) parts of the nucleus. In order to compare different markers we analyzed thin parallel sections of the same embryo. The markers selected were Choline Acetyl Transferase (ChAT) and Tyrosine Hydroxylase (TH), to identify the oculomotor nucleus (III) and the substantia nigra pars compacta (SNC) respectively. *Gad2* and *vGluT2* were studied to delimit the GABAergic and glutamateric populations.* Cplx1* and *Npas1* were used to test the maturation of RN neurons.

**Figure 2:**
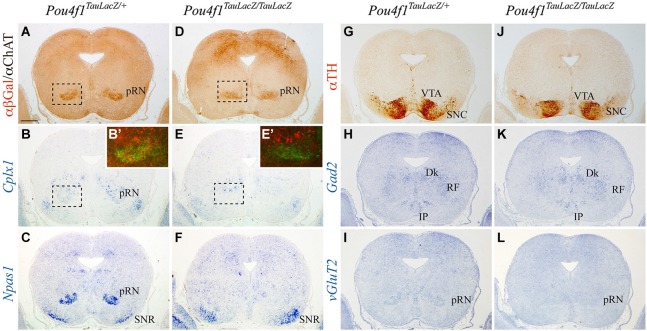
**Disorganization of pRN in absence of *Pou4f1***. Coronal diencephalic sections in *Pou4f^TauLacZ/+^* **(A–C, G–I)** and *Pou4f^TauLacZ/TauLacZ^* **(D–F, J–L)** embryos at E18.5 processed by immunohistochemistry **(A,D,G,J)** or *in situ* hybridization **(B,C,E,F,H,I,K,L)**. The *Pou4f ^TauLacZ/TauLacZ^* pRN showed a certain grade of disorganization in the diencephalic region as compared to the control **(A,D)**. The *Cplx1* expression was weakly detected in the control **(B)**, while the *Npas1* expression was detected in the pRN and the SNR **(C)**. In *Pou4f ^TauLacZ/TauLacZ^*, *Cplx1* and *Npas1* expression was completely absent in the pRN but remained unaffected in SNR (E, F). Squares shows overlapping and magnifications of these areas in **(B’)** and **(E’)**. The TH distribution was used to identify the SNC **(G,J)**. *Gad2* and *vGluT2* was used to identify GABAergic and glutamatergic neurons respectively **(H,I,K,L)** Abbreviations: Dk, Nucleus of Darkschewitsch; IP, Interpeduncular nucleus; RF, reticular formation; pRN, parvocellular red nucleus; SNC, substantia nigra pars compacta; SNR, substantia nigra pars reticulata; VTA, ventral tegmental area. Scale bar = 450 μm.

The pRN is located in the diencephalic basal plate (Figure 2A), confirmed by the absence of ChAT positive neurons. In the control, *Cplx1* was weakly expressed in the pRN while *Npas1* was strongly expressed (Figures 2B,B’,C). The substantia nigra pars reticulata (SNR) was also positive for *Npas1* (Figure 2C). The identification of the SNR was confirmed by the location of the SNC and ventral tegmental area (VTA) (Figure 2G). We recognized GABAergic populations as the Darkschewitsch nucleus, the reticular formation (RF) and the Interpeduncular nucleus (Figure 2H). *vGluT2* allowed us to identify the glutamatergic neurons of the pRN (Figure 2I). In the mutant, the pRN was dispersed and closer to the midline (Figure 2D). Both, *Cplx1* and *Npas1* were almost not detectable in the area of the pRN (Figures 2E,E’,F). The SNR was still positive for *Npas1* (Figure 2F) and its location was also verified by the presence of the dopaminergic neurons of the SNC (Figure 2J). The GABAergic populations did not display any obvious alteration (Figure 2K) meanwhile the glutamatergic neurons of the pRN could not be detected (Figure 2L).

**Figure 3:**
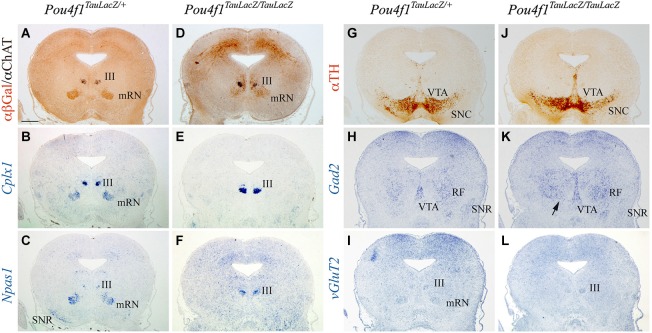
**Disorganization of mRN in absence of *Pou4f1***. Coronal mesencephalic sections in *Pou4f^TauLacZ/+^* **(A–C, G–I)** and *Pou4f^TauLacZ/TauLacZ^* **(D–F, J–L)** embryos at E18.5 processed by immunohistochemistry **(A,D,G,J)** or *in situ* hybridization **(B,C,E,F,H,I,K,L)**. The *Pou4f^TauLacZ/TauLacZ^* mRN exhibited a clear spatial disorganization while the III is completely normal **(A,D)**. *Cplx1* expression identified both mRN and III in *Pou4f1 ^TauLacZ/+^* **(B)**. In the mutant, *Cplx1* was exclusively expressed in the III **(E)**. The *Npas1* expression identified the same populations **(C)**. *Npas1* was lost in the mutant mRN **(F)**. The TH distribution was used to identify the SNC **(G,J)**. *Gad2* and *vGluT2* was used to identify GABAergic and glutamatergic neurons respectively **(H,I,K,L)**. Abbreviations: III, oculomotor complex; mRN, magnocellular red nucleus; RF, mesencephalic reticular formation; SNC, substantia nigra pars compacta; SNR, substantia nigra pars reticulata; VTA, ventral tegmental area. Scale bar = 450 μm.

The mRN located in the midbrain basal plate coincided with ChAT positive motor neurons of the III (Figure 3A). The expression of *Cplx1* in the mRN was stronger than in the pRN and the III was also intensively positive (Figure 3B). *Npas1* was also expressed in both populations and in the SNR (Figure 3C). The distribution of TH allowed us to identify the location of the SNC and VTA (Figure 3G). GABAergic neurons are concentrated in the mesencephalic RF, the SNR and the VTA (Figure 3H). The mRN and III appeared positive for *vGluT2* (Figure 3I). In the mutant, the mRN neurons were scattered and did not form a compacted population. The III did not display any obvious alteration (Figure 3D). *Cplx1* and *Npas1* were completely lost in the mRN, nevertheless their expression in the III was unaffected (Figures 3E,F). The *Cplx1* expression differences observed between control and mutant in the III is due to slight anteroposterior divergences between the sections. The SNC and VTA displayed a normal distribution (Figure 3J). The GABAergic neurons were present in the mesencephalic RF and SNR (Figure 3K). Nevertheless, the GABAergic neurons of the mesencephalic RF occupied the area of the absent mRN (arrow in Figure 3K). Finally, the v*GluT2* distribution confirmed the mRN absence and the maintenance of the III (Figure 3L).

In conclusion, the selected maturation markers, *Cplx1* and *Npas1*, were lost selectively in the pRN and mRN. These results suggest alterations in the final steps of RN neuronal development.

### Neuronal migration defects and RN cell death

**Figure 4:**
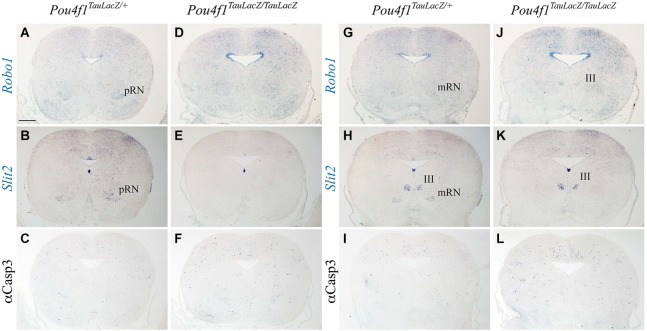
**Neuronal migration defects and cell death**. Coronal diencephalic **(A–F)** and mesencephalic **(G–L)** sections in *Pou4f^TauLacZ/+^* **(A–C, G–I)** and *Pou4f^TauLacZ/TauLacZ^* **(D–F, J–L)** embryos at E18.5 processed by *in situ* hybridization **(A,B,D,E,G,H,J,K)** or immunohistochemistry **(C,F,I,L)**. The *Robo1* and *Slit2* expression identified the pRN **(A,B)** and the mRN and III **(G,H)**. The *Robo1* and *Slit2* expression is completely lost in the mutant in both pRN and mRN **(D,E,J,K)**. Caspase3 do not show any difference between control and mutant **(C,F,I,L)**. Abbreviations: III, oculomotor complex; mRN, magnocellular red nucleus; pRN, parvocellular red nucleus. Scale bar = 450 μm.

We observed several alterations in the radial migration of the RN neurons in the absence of *Pou4f1*. Previous studies suggested a role of *Robo1* and *Slit2* in this process (Prakash et al., 2009). We analyzed the expression of these molecules in the two RN subnuclei. *Robo1* was expressed in the ventricular layer of the alar plate and in the pRN (Figure 4A) and in the mRN and III (Figure 4G). *Slit2* was expressed in the ventricular layer of the floor plate and in the basal mantel layer we detected the pRN (Figure 4B), the mRN and the III (Figure 4H). In the mutant, *Robo1* and *Slit2* displayed the same distribution being both non-detected in the presumptive area of the RN (Figures 4D,E,J,K). In the first description of the *Pou4f1* lack of function (McEvilly et al., 1996; Xiang et al., 1996) it was described the death of the RN neurons in the last stages of embryonic development. With the aim to confirm this event we analyzed the distribution of Caspase3 (marker of apoptotic neurons). We did not find any significant increment of this marker in the diencephalic (Figures 4C,F) or in the mesencephalic territory (Figures 4I,L). Therefore, the selective lost of both *Robo1* and *Slit2* may play a role in the altered distribution of the RN neurons.

### Defects in descending rubrospinal tract

**Figure 5:**
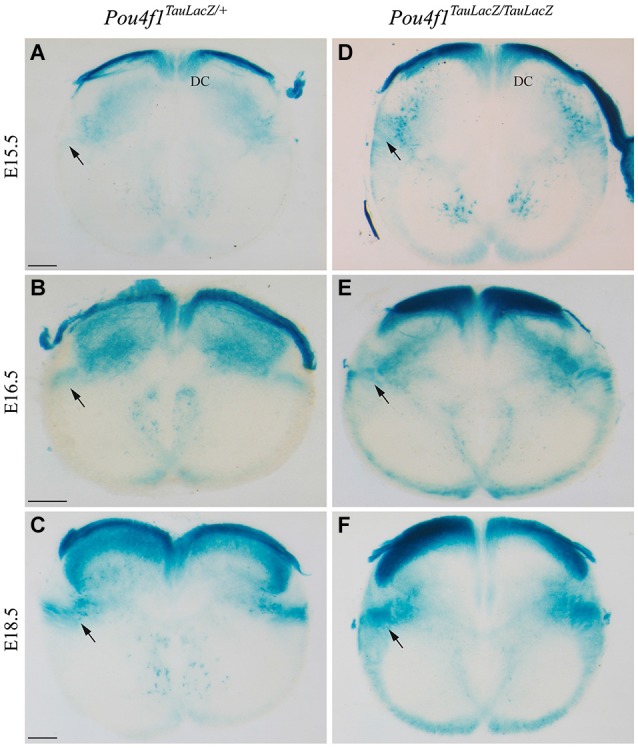
**The RST in the spinal cord. Abnormal growth in absence of *Pou4f1***. Coronal sections of cervical spinal cord in *Pou4f^TauLacZ/+^* **(A–C)** and *Pou4f^TauLacZ/TauLacZ^* **(D–F)** embryos processed by X-Gal staining. We have analyzed the embryonic stages: E15.5 **(A,D)**, E16.5 **(B,E)** and E18.5 **(C,F)**. The pioneer axons of the RST were detected for the first time at E15.5 (arrow in **(A)**). From E16.5 onwards, the RST was well established in the dorso-lateral region of the spinal cord (arrow in **(B,C)**). In the mutant the RST was also observed from E15.5 onwards but it occupied a wider region in the spinal cord (arrow in **(D,E,F)**). Abbreviations: DC, dorsal column. Scale bars = 150 μm.

To unveil the effect of altered maturation of RN projections, we analyzed the development of the descending portion of the RST. As described above, the first portion of the RST was generated normally in the mutant embryos. After the vtg, the RST descends through the spinal cord in a dorsolateral position. In control embryos, at E15.5 the RST was almost not visible at cervical levels of the spinal cord (arrow in Figure 5A). One day later, at E16.5, the tract was easily identified (arrow in Figure 5B). Finally, at E18.5, the RST displayed a dense labeling in control embryos (arrow in Figure 5C). In mutant embryos, at E15.5 the presumptive area of the RST displayed a clear labeling (probably due to the LacZ double copy; arrow in Figure 5D). At E16.5, the distribution of LacZ positive fibers was wider than in the control (arrow in Figure 5E). Finally, at E18.5, the tract exhibited an irregular distribution and an abnormal axonal termination area (arrow in Figure 5F).

Hence, the altered maturation of the RN neurons is translated into an anomalous distribution of the descending RST in the spinal cord.

## Discussion

### *POU4F1* role in red nucleus differentiation and maturation

*Pou4f1* is expressed during embryonic development and adulthood in the RN defining its identity. This transcription factor is integrated in the genetic cascade necessary to specify the RN neurons. This cascade is triggered by the positional information emitted by the secondary organizers of the neural tube (the isthmic organizer and the floor plate in this case; Echevarría et al., 2003). Nevertheless it was proven that *Pou4f1* is independent of *Sonic Hedgehog* direct induction (Perez-Balaguer et al., 2009). Previous studies described that the absence of this transcription factor prevents the normal specification and leads to a loss of posterior RN neurons (Fedtsova and Turner, 1995; McEvilly et al., 1996; Xiang et al., 1996; Agarwala and Ragsdale, 2002). The use of the transgenic line *Pou4f1^TauLacZ/TauLacZ^* allowed us to confirm the initial specification of this population in the *Pou4f1* loss-of-function embryos. The RN was clearly disorganized during embryonic development. However we did not observe a posterior loss of the nucleus. The RN has two different subpopulations originated in adjacent territories, the pRN in the diencephalon and the mRN in the midbrain where it coincides with III (Massion, 1967; Liang et al., 2012a,b). In *Pou4f1^TauLacZ/TauLacZ^* mice, the RN was completely disorganized being this phenotype more pronounced in the mRN.

*Npas1* and *Cplx1* are expressed in both RN and III in the mes-diencephalic basal plate. The characterization of their expression in the absence of *Pou4f1* results interesting in order to unveil the involvement of these transcription factors in the maturation of RN neurons. *Npas1* was proposed to cause an environmental change from a proliferative to differentiation stage of neuronal progenitors (Liu et al., 1994; Studer et al., 2000; Shingo et al., 2001; Ohsawa et al., 2005). *Cplx1* plays an important role in the modulation of neurotransmitter release. Its presence indicates a proper synaptic maturation (Glynn et al., 2005, 2007; Kielar et al., 2012; Gispert et al., 2014).

*Npas1* is expressed equally in the pRN and mRN and could be involved in maturation processes of the whole nucleus. Focusing on the diencephalic region, the pRN is comprised partially by small GABAergic neurons (Liang et al., 2012b). This transcription factor is also expressed in inhibitory neurons of other neuronal regions such as the hippocampus, dentate gyrus and some cortical layers (Zhou et al., 1997; Taylor and Zhulin, 1999; Rutter et al., 2001; Erbel-Sieler et al., 2004) and it is involved in their proper development (Zhao et al., 2008). In *Pou4f1^TauLacZ/TauLacZ^* mice, *Npas1* expression was completely lost in both the pRN and mRN. The lack of this transcription factor could partially account for the maturation phenotype observed in the RN neurons.

The expression of *Cplx1* is not homogeneous along the RN nucleus, it is weakly expressed in the pRN and strongly expressed in the mRN. This indicates different requirements of this transcription factor in the diencephalic and mesencephalic regions, which might be related to the different synaptic nature of these two RN components. *Cplx1* expression in the *Pou4f1* loss-of-function embryos was completely lost in the pRN and mRN. This could contribute to an abnormal final maturation, innervation and synaptic contact of the RST as it has been described for other axonal projections affected in absence of *Pou4f1* (Eng et al., 2001). The selective loss of *Cplx1* and *Npas1* in *Pou4f1^TauLacZ/TauLacZ^* RN suggests alterations in maturation and synaptogenesis of their neurons.

We also observed a strong delay in the radial migration of the RN neurons and a wider final distribution in the mantle layer. The *Robo1/Slit2* guidance mechanism is lost in the absence of *Pou4f1* in the RN and they could contribute to the phenotype observed as it was suggested for the III and trochlear nuclei (Prakash et al., 2009). Surprisingly, we did not corroborate the described cell death of the RN in the last stages of embryonic development (McEvilly et al., 1996; Xiang et al., 1996).

### Disorganized rubrospinal tract in the spinal cord

We have also described the time window in which the axonal fibers of the RST reach their destination. At E12.5, the pioneer axons of the RST leave the RN and 3 days later these fibers have traveled along the hindbrain to reach the cervical spinal cord.

At E18.5 the RST should be completely developed because it is critical for establishing rudimental motor skills (Williams et al., 2014). At this stage, the RST together with other spinal cord longitudinal axonal tracts showed certain disorganization in the absence of *Pou4f1*. The dorsal root ganglia of the spinal cord are *Pou4f1* positive and they are also strongly affected in this mutant, this could also contribute to the RST altered distribution. The disorganization of the tract and the loss of *Cplx1* and *Npas1* in the RN could indicate a failure in the proper maturation, motor function development, innervation and synaptogenesis of this population in the *Pou4f1^TauLacZ/TauLacZ^* mice. This defect could in turn cause the altered movements observed in postnatal *Pou4f1^TauLacZ/TauLacZ^* or *Cplx*^−/−^ deficient mice (Xiang et al., 1996; Glynn et al., 2005, 2007; Kielar et al., 2012; Gispert et al., 2014).

In conclusion, *Pou4f1* is not necessary for the generation of the RN but is required for the maintenance and maturation of its neurons and thus for the proper development of the RST.

## Author contributions

All authors had full access to all the data in the study and take responsibility for the integrity of the data and the accuracy of the data analysis. Conceived and designed the experiments: Jesus E. Martinez-Lopez, Salvador Martinez and Eduardo Puelles; Performed the experiments: Jesus E. Martinez-Lopez, Juan A. Moreno-Bravo and M. Pilar Madrigal; Analyzed the data: Jesus E. Martinez-Lopez and Eduardo Puelles; Wrote the article: Jesus E. Martinez-Lopez and Eduardo Puelles; Obtained funding: Salvador Martinez and Eduardo Puelles.

## Conflict of interest statement

The authors declare that the research was conducted in the absence of any commercial or financial relationships that could be construed as a potential conflict of interest.
